# Effects of replacing of alfalfa hay with *Plantago lanceolata* hay on digestibility, methane production and microbial protein production of total mixed diet

**DOI:** 10.1007/s11250-024-04017-8

**Published:** 2024-05-10

**Authors:** Bilal Selcuk, Yakup Bilal, Tugba Bakir, Cagri Ozgur Ozkan

**Affiliations:** Department of Animal Science, Kahramanmaraş Sütçü İmam University, Kahramanmaraş, Türkiye

**Keywords:** Digestibility, Partitioning factor, *Plantago lanceolata*, Methane, Microbial protein

## Abstract

The aim of current experiment was to determine the effect of replacement of alfalfa hay with ribwort plantain (*Plantago lanceolata*) hay in ruminant diets on the fermentation parameters such as gas production, methane (CH_4_) production, true digestible dry matter (TDDM), true digestibility (TD), partitioning factor, microbial protein, and efficiency of microbial protein using in vitro gas production technique. The alfalfa hay was replaced with *P. lanceolata* hay in a diets isocaloric (2650 kcal/kg DM) and nitrogenic (17% CP kg DM) at the ratio of 0, 5, 10 and 15%. Partial substitution of alfalfa hay with *P. lanceolata* hay had no significant effect on gas and methane (ml/incubated substrate or %) production whereas the partial substitution had a significant effect on TDDM, TD, gas (ml/digested DM), CH_4_ (ml ml/digested DM) and microbial MP of diets. The replacement of alfalfa hay with ribwort plantain hay shifted the fermentation pattern from gas and methane production to microbial protein production. Therefore alfalfa hay can be replaced with ribwort plantain hay with high digestibility and anti-methanogenic potential in ruminant diets up to 15% to decrease methane production and improve microbial protein production. However further in vivo experiments are required to determine the effect of replacement on feed intake and animal production.

## Introduction

Forage is one of the important components of ruminant diets for rumination, digestive physiology and production (Van Soest et al. 1991). Recently *Plantago lanceolata* has drawn considerable attention due to its nutritional composition and anti-methanogenic potential for ruminants (Della Rosa et al. 2022; Kara et al. 2015; Kara et al. 2016; Ekinci et al. 2018; Kara et al. 2018; Bariroh et al. 2021). *Plantago* L is a perennial plant with its primary homeland being determined as Europe and Siberia; it is widely distributed throughout many parts of the world, and its flowering period occurs between April and September (Ulcay and Şener, 20202020). *Plantago lanceolata*, narrow-leaved plant, is one of over 250 spices of genus *Plantago* L (Hassemer 2019; Hassemer et al. 2019). *Plantago lanceolata* hay might provide animal with considerable amount protein and energy (Kara et al. 2018). Ribwort plantain also contains various bioactive compounds (Pacheco and Waghorn, 2008; Tamura and Nishibe, 2002), which may affect rumen microorganisms and fermentation parameters (Swainson, 2006; Yusuf et al. 2013; Rira et al. 2015; Sirohi et al. 2013). However, the effects of bioactive compounds on fermentation and digestibility when included in ruminant diets are unclear. Ruminant animals produce methane (CH_4_) due to fermentation in the rumen (Swain et al. 2016). The methane production not only results in energy loss (2–12% of the digestible energy) (Johnson and Johnson, 1995; Kaya et al. 2012) but also contributes global warming (IPCC, 2001). Therefore reducing methane production in combination with increasing microbial protein production in the rumen is one of the main goals of ruminant nutritionists. Recently Menke gas production technique (Menke et al. 1979) has been used to determine the effect of replacement of forages and concentrates in ruminant diets on fermentation parameters due to possible interaction among feed ingredients in diets (Metzler-Zebeli, 2012; Boga et al. 2022; Gemalmaz and Bilal, 2016; Vibart et al. 2009; Burke et al. 2000).

The aim of current experiment was to determine the effect of replacement of alfalfa with ribwort plantain hay in ruminant diets on the fermentation parameters such as gas production, methane production, true digestibility, partitioning factor, microbial protein, and efficiency of microbial protein.

## Materials and methods

The feed ingredients were obtained from a commercial feed factory in Kahramanmaraş province. Ribwort plantain was harvested from pastures in Kahramanmaraş province in May at the blooming period. The feed ingredients were dried in an oven at 105° C for 2 h (AOAC, 1990). The samples were ground through a 1 mm sieve. Rumen fluid was obtained from a slaughterhouse in Kahramanmaras province from 3 Awassi sheep. The sheeps were fed a mixture of alfalfa hay and barley in a 60 − 40% ratio. The rumen fluid was obtained from the sheep with 55–62 kg weight and transferred to the laboratory in a thermos flasked CO_2_ in 30 min.

### Chemical analyses

The dry matter (DM)(ID: 950.05), ether extract .(EE)(ID: 920.39), crude ash (CA)(ID: 942.05), and crude protein (CP)(ID: 976.05) contents of the feed ingredients were determined by the method reported by AOAC (1990). The diets were prepared to be isocaloric and isonitrogenous, containing 2650 kcal/kg DM of metabolic energy (ME) and 17% crude protein (CP) of kg DM, according to the requirements reported by NRC (2007) for lambs. The mixture proportions of the diets are given in Table 1.

**Table 1:** The composition of experimental the diets (g)

Substitution rate (%)				
	0	5	10	15
Alfalfa hay	400	350	300	250
Ribwort plantain hay	-	50	100	150
Oil	20	20	20	20
Wheat bran	110.09	77.24	44.40	11.56
Sunflower meal	120.07	136.17	152.25	168.34
Barley	323.84	340.59	357.35	374.10
Salt	10	10	10	10
Calcium carbonate	15	15	15	15
Min-Vit Mix	1	1	1	1
Total (g)	1000	1000	1000	1000
ME (kcal/kg DM)	2650	2650	2650	2650
CP (%)	17	17	17	17
EE (%)	3.76	3.73	3.95	4.05
Ash (%)	5.98	6.13	6.28	6.40

ME: Metabolisable Energy, CP: Crude protein calculated. EE: Ether extract calculated. Ash: ash calculated.

The gas production of the rations utilized in the study was assessed using the in vitro gas production method developed by Menke et al. (1979). Approximately 0.5 g of sample were transferred into 100 ml of glass syringes and subjected to fermentation in buffered rumen fluid for 24 h. Rumen fluid at a 1:2 ratio was buffered in an anaerobic environment. The buffer consist of 474 ml of distilled water, 237.33 ml macro mineral solution, 237.33 ml of buffer solution, 0.12 ml of trace mineral solution, 1.22 ml of resazurine and 50 ml of reducing solution. The methane CH_4_ content of the gas produced was measured with an infrared methane analyzer (Goel et al. 2008). The true dry matter digestibility (TDDM) content of the rations was determined by the method described by Blümmel et al., (1997). The metabolic energy (ME) and organic matter digestibility (OMD) of the feedstuffs were calculated according to the method described by Menke and Steingass (1988).

$$\text{M}\text{E} (\text{M}\text{J}/\text{k}\text{g} \text{d}\text{m}) = 1.68 + 0.1418\text{G}\text{P} + 0.073\text{C}\text{P} + 0.217\text{E}\text{E} - 0.028\text{C}\text{A}$$

$$\text{O}\text{M}\text{D} \left(\text{\%}\right) =14.88 + 0.8893\text{G}\text{P} + 0.448\text{C}\text{P} + 0.651\text{C}\text{A}$$

GP: 24-hour incubation results in gas production in the samples (200 mg/DM).

CP: Crude protein (%)

EE: Ether extract (%)

CA: Crude ash (%)

True digestible dry matter (TDDM); true digestibility (TD); partitioning factor (PF); microbial protein production (MP) and efficiency of microbial protein (EMP) values have been calculated using the formulas reported by Blümmel et al. (1997) and Vercoe et al. (2010).

$$\text{T}\text{D}\text{D}\text{M} = \text{D}\text{r}\text{y} \, \text{M}\text{a}\text{t}\text{t}\text{e}\text{r} \,\text{I}\text{n}\text{c}\text{u}\text{b}\text{a}\text{t}\text{e}\text{d} \left(\text{m}\text{g}\right) - \text{R}\text{e}\text{m}\text{a}\text{i}\text{n}\text{i}\text{n}\text{g} \, \text{D}\text{r}\text{y} \, \text{M}\text{a}\text{t}\text{t}\text{e}\text{r} \left(\text{m}\text{g}\right)$$

$$\text{T}\text{D} \left(\text{\%}\right) = (\text{T}\text{D}\text{D}\text{M} / \text{D}\text{r}\text{y} \, \text{M}\text{a}\text{t}\text{t}\text{e}\text{r} \, \text{I}\text{n}\text{c}\text{u}\text{b}\text{a}\text{t}\text{e}\text{d}) \, \text{x} \, 100$$

$$\text{P}\text{F} = (\text{T}\text{D}\text{D}\text{M} / \text{G}\text{a}\text{s} \, \text{P}\text{r}\text{o}\text{d}\text{u}\text{c}\text{t}\text{i}\text{o}\text{n})$$

$$\text{M}\text{P} = (\text{T}\text{D}\text{D}\text{M} - (2.2 \text{x} \text{G}\text{a}\text{s} \, \text{P}\text{r}\text{o}\text{d}\text{u}\text{c}\text{t}\text{i}\text{o}\text{n}\left)\right)$$

$$\text{E}\text{M}\text{P} = \left(\right((\text{T}\text{D}\text{D}\text{M} - (2.2 \text{x} \text{G}\text{a}\text{s} \, \text{P}\text{r}\text{o}\text{d}\text{u}\text{c}\text{t}\text{i}\text{o}\text{n}\left)\right)/\text{T}\text{D}\text{D}\text{M}) \, \text{x} \, 100$$

The chemical compositions of the feed ingredients used in the preparation of the rations, GP, ME, and OMD values are given in Table 2.

**Table 2:** Chemical compositions, gas production, metabolisable energy and organic matter digestibility values of feed raw materials

Ingredients	DM (%)	CA (%)	EE (%)	CP (%)	GP(ml)	ME(MJ/kgDM)	OMD (%)
Alfalfa	94.41	9.41	1.53	18.64	47.70	9.87	71.78
Ribwort Plantain	92.75	11.37	4.03	12.85	56.67	10.84	78.43
Wheat Bran	91.12	3.41	2.69	15.53	66.24	12.69	82.97
Sunflower meal	92.44	7.18	1.45	39.07	40.05	10.33	72.67
Barley	90.78	3.03	2.12	11.25	61.80	11.64	76.85

DM: Dry Matter. CA: Crude Ash. EE: Ether extract. CP: Crude Protein. GP: Gas production, ME: Metabolic Energy. OMD: Organic Matter Digestibility.

### Statistical analysis

The statistical analysis of the data obtained in the study was performed by one-way variance (ANOVA) analysis in SPSS 20.0 package program (SPSS 2011). The differences between the means were determined by Duncan multiple comparison test (Duncan, 1955).

## Results

The effect of partial substation of alfalfa hay with ribwort plantain hay on fermentation parameters and microbial protein values are given in Table 3. Partial substitution of alfalfa hay with ribwort plantain hay had no significant effect on gas and methane (ml/incubated substrate or %) production whereas the partial substitution had a significant effect on TDDM, TD, gas (ml/digested DM), CH_4_ (ml ml/digested DM) and MP of diets.

**Table 3:** The effect of partial substitution of alfalfa hay with ribwort plantain ha y on fermentation parameters and microbial protein values of rations

	Substitutions rate (%)					
	0	%5	%10	%15	SEM	*p*
Gas(ml)/Incubated	99.75	98.50	97.75	98.00	1.952	0.745
CH_4_(ml)/Incubated	15.32	15.21	15.14	14.15	0.560	0.188
CH_4_ (%)	15.36	15.45	15.49	14.43	0.506	0.174
TDDM(mg)	348.64^a^	358.33^b^	360.47^b^	362.11^b^	3.010	0.003
TD (%)	73.65^a^	75.39^b^	76.87^c^	76.53^bc^	0.626	0.010
Gas(ml)/g digested DM	286.12^a^	274.89^b^	271.19^b^	270.68^b^	2.275	0.000
CH_4_(ml)/g digested DM	43.94^a^	42.45^b^	42.00^b^	39.08^c^	0.340	0.000
PF	3.49	3.64	3.68	3.69	0.077	0.080
MP (mg)	129.19^a^	141.63^b^	145.42^b^	146.51^b^	5.207	0.022
EMP (%)	37.05^a^	39.52^ab^	40.32^b^	40.44^b^	1.283	0.072

^abc^There is no difference between averages with the same symbol and in the same row. SEM: Standart Error Mean (*P* < 0.05). GP: Gas Production 500 mg DM. CH4 (ml) and CH4 (%): Methane production amount of rations 500 mg DM. TD: True digestibility. TDDM: True digestible dry matter 500 mg DM. PF: Partitioning factor. MP: Microbial protein. EMP: Efficiency of microbial protein

The TDDM (mg), TD (%) and MP ranged from 348.64 to 362.11 mg and 73.65 to 76.87% respectively. The replacement of alfalfa hay with ribwort plantain hay significantly increased the TDDM (g) and TD (%) On the other hand, the replacement of alfalfa hay with ribwort plantain hay resulted in the reduction in gas production (ml /digested DM) and CH_4_ production (ml/digested DM) in a diets. The gas production (ml /digested DM) and CH_4_ production (ml/digested DM) ranged from 270.68 to 286.12 ml and 39.08 to 43.94 ml respectively. In addition the replacement of alfalfa hay with ribwort plantain hay resulted in an increase in MP of diets.

## Discussion

As can be seen from Table 3 the gas production resulted from the fermentation of carbohydrates is presented as gas production/incubated or gas production/digested. There is no significant effect of replacing alfalfa hay with ribwort plantain hay on gas production/incubated whereas replacement had a significant effect on gas production/digested DM. Therefore, a comparison of diets in terms of gas production would be misleading when gas production/ incubated sample. The presentation of gas production as a gas production/digested DM is more informative and meaningful. The incubated substrate can be diverted into gas or microbial protein production. The gas production is generally though as a wasteful process of fermentation. It is interesting to notice that there is no significant differences in gas production/incubated sample whereas microbial protein production increased due to replacement of alfalfa hay with ribwort plantain hay. It is very difficult to explain the increase in microbial protein when the gas production/incubated sample is used. This situation may be attributed to the specific rumen microbiota in use. For instance, in a conducted study, despite oregano plant and alfalfa sharing similar chemical compositions, oregano plant exhibited distinct rumen kinetics and degradability from alfalfa in dairy cows due to differences in rumen microbiota (Gultepe et al. 2020). On the other hand, it is very easy and clear to understand the possible reason why the increase in microbial protein production occurs when the gas production is represented as gas production/digested or fermented DM is used in the current experiment. Therefore it has been recommended to represent the gas produced during fermentation as gas production/digested dry matter more reasonable and meaningful than gas production/incubated substrate (Navarro-Villa et al. 2011; Yanez-Ruiz et al. 2016).

As can be seen from Table 3 the TDDM of diets increased with replacement of alfalfa hay with ribwort plantain hay. The increase in TDDM of diets is possibly associated with high organic matter of ribwort plantain hay indicated in Table 2. The OMD value of ribwort plantain hay was considerably higher than that of alfalfa hay. It can be expected that the replacement of alfalfa hay with ribwort plantain hay with high OMD value would increase the TDDM of diets. However, the replacement of alfalfa hay with ribwort plantain hay did not increase the gas production.

The replacement of alfalfa hay with ribwort plantain hay provided micro-organism with more digestible substrate with less gas production resulted in high microbial protein production. It is evident that the replacement of alfalfa hay with ribwort plantain hay shifted the fermentation pattern from gas production to microbial production. The shift in fermentation patter is desirable to improve the efficiency of feed utilization in the rumen. Generally the gas production during the fermentation results in decrease in the efficiency of feed utilization. The more gas produced during fermentation the less efficient of feed utilization. It is well know that microbial protein is an important part of metabolisable protein used by ruminant animals to meet their protein requirement for both maintenance and production (Das et al. 2014; Kamalak et al. 2005).

As in the presentation of gas production, the presentation of methane production is also very important. As can be seen from Table 2, there is no significant differences among diets in terms of methane production when methane represent as methane/incubated substrate or percentage whereas there is a significant differences among diets in terms of methane when methane production was represented as methane/digested substrate. It is evident that replacement of alfalfa hay with ribwort plantain hay significantly reduced the methane production of diets. The reduction of methane production due to replacement of alfalfa hay with ribwort plantain hay is possibly associated with carbohydrate compositions and bioactive seconder metabolites in ribwort plantain. However, it would be very informative if carbohydrate compositions and seconder metabolites of ribwort plantain hay had been analyzed in the current experiment. This is one of limitations of current experiment to explain the decrease in the methane production of diets.

The replacement of alfalfa hay with ribwort plantain hay shifted the fermentation pattern from gas and methane production to microbial protein production. Therefore alfalfa hay can be replaced with ribwort plantain hay with high digestibility and anti-methanogenic potential in ruminant diets up to 15% to decrease methane production and improve microbial protein production. However further in vivo experiments are required to determine the effect of replacement on feed intake and animal production.

## Data Availability

All relevant data are within the manuscript and the datasets generated and/or analyzed during this study are available from the corresponding author on reasonable request.
